# Behavioral Sensitization to the Disinhibition Effect of Ethanol Requires the Dopamine/Ecdysone Receptor in *Drosophila*

**DOI:** 10.3389/fnsys.2017.00056

**Published:** 2017-08-02

**Authors:** Gissel P. Aranda, Samantha J. Hinojos, Paul R. Sabandal, Peter D. Evans, Kyung-An Han

**Affiliations:** ^1^Neuromodulation Disorders Cluster at Border Biomedical Research Center, Department of Biological Sciences, University of Texas at El Paso El Paso, TX, United States; ^2^The Inositide Laboratory, The Babraham Institute Cambridge, United Kingdom

**Keywords:** dopamine, behavioral sensitization, courtship disinhibition, mushroom body, ethanol, tolerance, D1 receptors, DopEcR

## Abstract

Male flies under the influence of ethanol display disinhibited courtship, which is augmented with repeated ethanol exposures. We have previously shown that dopamine is important for this type of ethanol-induced behavioral sensitization but the underlying mechanism is unknown. Here we report that DopEcR, an insect G-protein coupled receptor that binds to dopamine and steroid hormone ecdysone, is a major receptor mediating courtship sensitization. Upon daily ethanol administration, *dumb* and *damb* mutant males defective in D1 (dDA1/DopR1) and D5 (DAMB/DopR2) dopamine receptors, respectively, showed normal courtship sensitization; however, the DopEcR-deficient *der* males exhibited greatly diminished sensitization. *der* mutant males nevertheless developed normal tolerance to the sedative effect of ethanol, indicating a selective function of DopEcR in chronic ethanol-associated behavioral plasticity. DopEcR plays a physiological role in behavioral sensitization since courtship sensitization in *der* males was reinstated when DopEcR expression was induced during adulthood but not during development. When examined for the DopEcR’s functional site, the *der* mutant’s sensitization phenotype was fully rescued by restored DopEcR expression in the mushroom body (MB) αβ and γ neurons. Consistently, we observed DopEcR immunoreactivity in the MB calyx and lobes in the wild-type *Canton-S* brain, which was barely detectable in the *der* brain. Behavioral sensitization to the locomotor-stimulant effect has been serving as a model for ethanol abuse and addiction. This is the first report elucidating the mechanism underlying behavioral sensitization to another stimulant effect of ethanol.

## Introduction

Fruit flies are routinely exposed to ethanol in fermented fruits and food. In a laboratory setting, ethanol causes many behavioral responses that include hyper-locomotor activity, disinhibition, loss of motor control and sedation. Specifically, low doses of ethanol increase walking speed and turning, low to moderate doses induce disinhibited sexual activity and high doses lead to loss of postural control and sedation (Bainton et al., 2000; Lee et al., 2008). Flies develop tolerance to the sedative effect when repeatedly exposed to ethanol (Scholz et al., 2000; Lee et al., 2008). These observations indicate that ethanol-induced behaviors in flies and intoxicated humans are similar; thus, the knowledge of their neurobiological basis could help not only uncover evolutionarily conserved vs. distinct neural, cellular and molecular pathways but also gain insight into effective intervention of ethanol abuse and addiction. The biogenic amine dopamine is involved in locomotor stimulating and rewarding effects of ethanol in flies, rodents and humans (Devineni and Heberlein, 2013; Abrahao et al., 2014; Jayaram-Lindström et al., 2016). For example, ethanol intake elevates extracellular dopamine levels in the nucleus accumbens in rodents (Meyer et al., 2009; Vena et al., 2016). Likewise in flies, blockade of dopamine biosynthesis via 3IY that inhibits tyrosine hydroxylase dampens the ethanol’s locomotor stimulant effect, which is reversed by L-DOPA feeding (Bainton et al., 2000). D1 and D2 dopamine receptors are involved in the locomotor stimulating and rewarding effects of ethanol in rodents (Lê et al., 1997; Matsuzawa et al., 1999; Arias et al., 2010) while D1 receptor is involved in both effects in flies (Kong et al., 2010; Kaun et al., 2011).

Behavioral sensitization is an escalated response to repeated drug use and underlies drug abuse and addiction (Berridge and Robinson, 2016). Dopamine is also important for behavioral sensitization to the ethanol’s locomotor stimulant effect in rodents (Camarini and Pautassi, 2016). Repeated local or global ethanol treatments induce sensitized activity of dopamine neurons in the ventral tegmental area (Brodie, 2002; Ding et al., 2009). Pharmacological and genetic studies show involvement of both D1 and D2 family receptors in sensitization. For example, D1 and D3 knockout mice are defective in sensitization to chronic ethanol exposure (Harrison and Nobrega, 2009). Interestingly, D3 knockout mice develop normal sensitization to amphetamine, indicating the D3’s function in the selective sensitization pathway. Observations on D2 knockout mice are conflicting: one study (Harrison and Nobrega, 2009) shows defective sensitization whereas another study (Palmer et al., 2003) reveals enhanced sensitization when the knockout mice in the same genetic background are compared. Thus, only a particular environmental or treatment condition involves D2-mediated sensitization. Together these observations indicate that the dopamine system mediates multiple yet distinct sensitization processes. Similar to rodents, flies develop sensitization to the locomotor stimulant effect of ethanol (Kong et al., 2010) and the mechanism is yet to be determined.

A prominent effect of ethanol in humans is disinhibition. Disinhibited cognition and motor functions lead to risk taking behaviors and impulsivity, which facilitate ethanol or other substance abuse and addiction (Field et al., 2010; Dalley et al., 2011; Morris et al., 2016). However, the mechanism underlying ethanol-induced disinhibition remains poorly understood. We have previously shown that dopamine mediates ethanol-induced courtship disinhibition and behavioral sensitization to this effect in *Drosophila* (Lee et al., 2008). *Drosophila* has three D1 family receptors: dDA1/DopR1 D1; Sugamori et al., 1995), DAMB/DopR2 (D5; Han et al., 1996) and DopEcR (Srivastava et al., 2005). When stimulated by dopamine, DopEcR activates an increase in cAMP and the PI3 kinase pathway whereas ecdysone inhibits the effect of dopamine on cAMP and activates the MAP kinase pathway. Here we report that sensitization to the disinhibition effect of ethanol requires DopEcR function in the mushroom body (MB) neurons. The findings reported here provide a framework to unravel the relevant neural circuits and the cellular mechanisms.

## Materials and Methods

### *Drosophila* Strains and Culture

Flies were maintained on standard cornmeal agar medium at 25°C with 50% relative humidity under the 12 h light/12 h dark illumination condition. *Canton-S* was used as a wild-type strain. The DopEcR mutant used in this study is the insertion mutant *DopEcR*^c02142^ (also known as *DopEcR*^PB1^; hereafter *der*) generated by the Gene Disruption Project (FlyBase Consortium, 2003; Thibault et al., 2004) and has been previously described (FlyBase Consortium, 2003; Inagaki et al., 2012; Petruccelli et al., 2016). *der* was obtained from the Bloomington Stock Center (stock no. 10847) and backcrossed with Cantonized *w*^1118^ for six generations, and then the X chromosome was replaced with that of *Canton-S* to remove the *w*^1118^ mutation. *elav-GAL4* (stock no. 8765), *c739-GAL4* (stock no. 7362)*, c305a-GAL4* (stock no. 30829)*, UAS-mCD8-GFP* (stock no. 5137) and *PTRiP.JF03415* (stock no. 31981; FlyBase Consortium, 2003; Perkins et al., 2015) flies were obtained from the Bloomington Stock Center; *NP1131-GAL4* from Dr. Dubnau (Stony Brook University School of Medicine, Stony Brook, NY, USA); *fru^NP21-^GAL4* from Dr. Yamamoto (Tohoku University, Sendai, Japan); *NP225-GAL4* from Dr. Thum (University of Konstanz, Konstanz, Germany); *tub-GS-GAL4* from Dr. Kitamoto (University of Iowa, Iowa City, IA, USA); and *MB-GS-GAL4* from Dr. Roman (University of Houston, Houston, TX, USA). We have previously described *MB247-GAL4* and *MB247-GAL4, GAL80*^ts^ (Kim et al., 2007, 2013). *DopEcR* cDNA containing the open reading frame (Srivastava et al., 2005) was cloned under UAS in the gateway vector pTW (Akbari et al., 2009). The cloned receptor was injected into *w*^1118^ embryos, and germ-line transformed lines were outcrossed with Cantonized *w*^1118^ for six generations to normalize the genetic background and to remove potential second site mutations. Individual transgenes were placed in the *der* mutant background for rescue experiments. We previously reported the dDA1 (D1) mutant *dumb*^1^ and *dumb*^2^ (Kim et al., 2007) and the *damb* mutant defective in DAMB (D5; Cassar et al., 2015). For conditional rescue experiments involving the gene switch lines *MB-GS-GAL4* and *tub-GS-GAL4*, 10 mM RU486 (Mifepristone, M8046, Sigma-Aldrich, Saint Louis, MO, USA) was made in 80% ethanol and added to fly food to the final concentration of 500 μM. Flies were reared on the food containing RU486 for 1 day before and between ethanol exposures. All genotypes used for behavioral analyses including the controls (*Canton-S* and *der* mutants carrying only *GS-GAL4*) were fed with RU486 or vehicle for comparison.

### Immunohistochemical Analysis

The polyclonal DopEcR antibody was made commercially in a New Zealand white rabbit against the peptide GEPIHDKEYATALAEN that corresponds to the third cytoplasmic loop of the receptor (Pacific Immunology Corp, Ramona, CA, USA). Immunostaining was performed as previously described (Kim et al., 2013; Lim et al., 2014). Briefly, 4–5 day-old male brains were dissected in phosphate buffered saline (PBS) where the trachea around the brain was removed. Dissected brains were individually fixed with 4% PFA (paraformaldehyde and 0.04 M Lysine in PBS) at 4°C for 3 h and then rinsed three times in PBHT containing 0.5% Triton X-100 for 10 min each. Brains were solubilized in 1% Triton X-100 in PBHT for 1 h, incubated in the blocking solution (5% normal goat serum in PBHT) for 2 h and then incubated with the anti-DopEcR antibody (1:100 diluted in the blocking solution) at room temperature overnight. Brains were washed four times in PBHT for 1 h at room temperature and then overnight at 4°C before incubation with the goat Alexa 488-conjugated anti-rabbit IgG (Molecular Probes, Carlsbad, CA, USA) at room temperature for 2 h. After washes in PBHT, PBS and 0.12 M Tris-HCl, pH 7.4 (three times in each solution), brains were mounted in the VECTASHIELD medium (Vector Labs, Burlingame, CA, USA). Images were taken using the Zeiss LSM 700 confocal microscope (Carl Zeiss, Thornwood, NY, USA) and analyzed using the ImageJ software (NIH).

### Behavioral Tests

One to two-day-old males were collected under carbon dioxide (CO_2_) and aged in food vials for 2–3 days before tests. A group of 33 males was used as one data point in all behavioral tests. Ethanol exposure was performed in the Flypub consisting of a plastic chamber (57 mm D × 103 mm H) with the clear ceiling for videotaping behavior and the open bottom for administering ethanol as previously described (Lee et al., 2008). Flies were acclimated to the chamber for 10 min before ethanol exposure. A small petri dish containing a cotton pad applied with 1 ml of 95% ethanol was inserted to the bottom opening and flies were exposed to ethanol vapor till they were sedated. Four to six Flypubs were recorded together using a HD video camera (Q2F-00013 Microsoft LifeCam Studio, Redmond, WA, USA). The recorded movie files were used to score courtship activity. Flies were exposed to ethanol every 24 h for six consecutive days and were kept in food vials between exposures. The sedative effect of ethanol was measured by counting every 2 min the number of flies lying on their back or immobile for over 10 s. To obtain the mean sedation time (MST), the total sedation time, i.e., ∑(the number of sedated flies at each time interval × each time interval after ethanol administration, e.g., 2, 4, 6 and etc.), was divided by the total number of flies (Lee et al., 2008). Courtship activity consisting of singing (unilateral wing vibration), licking or attempted copulation (Baker et al., 2001) was monitored during 30 s (1 block) and the maximum number of flies engaged in courtship at a given time was scored. The average of 10 consecutive blocks (i.e., 5 min) giving the highest value was used to represent the percentage of males engaged in active intermale courtship per Flypub (Lee et al., 2008). Our earlier study (Lee et al., 2008) has shown that the maximal level of ethanol-induced courtship disinhibition is achieved on the exposure 4 or 5 and then maintained steady. Thus, we focus on exposure 1 for the initial level of ethanol-induced disinhibition, exposure 2 for sensitization induction and exposure 6 for maintenance in this study. The genotypes were blinded to the experimenters conducting ethanol exposure and scoring courtship or sedation.

### Data Analysis

Statistical analyses were performed using Minitab 16 (Minitab, State College, PA, USA) and JMP 13 (SAS, Cary, NC, USA). All data are reported as mean + or ± standard error of means (SEM). Normality was determined by the Anderson Darling goodness-of-fit test. Normally distributed data were analyzed by a two-tailed Student’s *t*-test or analysis of variance (ANOVA) with *post hoc* Tukey-Kramer HSD or Dunnett’s tests. Non-normally distributed data were analyzed by Kruskal-Wallis and *post hoc* Mann-Whitney tests.

## Results

### Tolerance to the Sedative Effect of Ethanol

**Figure 1:**
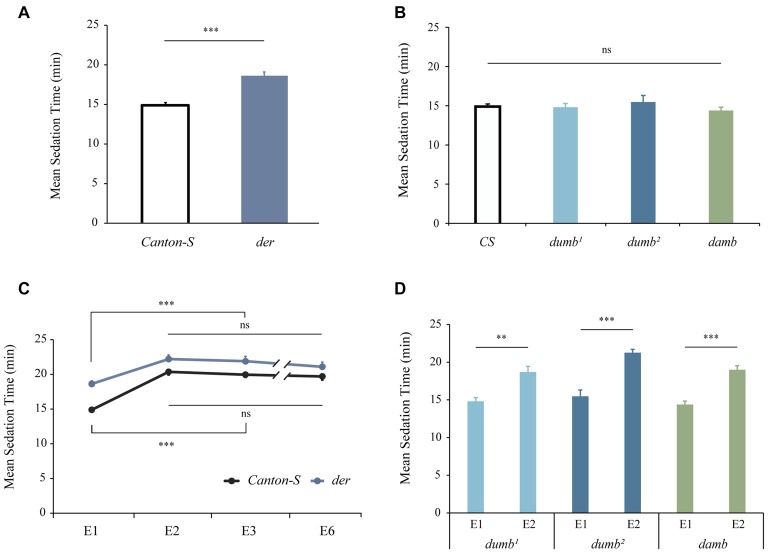
D1 family receptors are dispensable for ethanol tolerance. The wild-type *Canton-S* and D1 receptor mutants were exposed to ethanol and mean sedation time (MST) was measured. **(A)** *der* mutant males defective in DopEcR showed decreased sensitivity to the sedative effect of ethanol (***p < 0.0001 by two-tailed Student’s *t*-test; *Canton-S*, *n* = 26; *der*, *n* = 22). **(B)** *dumb*^1^and *dumb*^2^ mutant males defective in dDA1 (D1) and *damb* defective in DAMB (D5) exhibited normal sensitivity. Analysis of variance (ANOVA): *p* > 0.05, *n* = 8; ns, not significant. **(C)** *der* males showed normal tolerance development and maintenance to the sedative effect of ethanol. (***p < 0.0001 by ANOVA and *post hoc* Tukey-Kramer HSD tests; *Canton-S*, *n* = 26; *der*, *n* = 22). **(D)** *dumb* and *damb* males displayed normal tolerance. Student *t*-test; **p < 0.001; ***p < 0.0001; *n* = 8.

To investigate the roles of D1 family receptors in chronic ethanol effects, we employed the Flypub for mild ethanol delivery (Lee et al., 2008). We first measured the sedative effect of ethanol. Compared to the control *Canton-S* males, it took longer for *der* mutant males to get sedated (*p* < 0.0001; Figure 1A), demonstrating that *der* males defective in DopEcR have decreased sensitivity to the sedative effect of ethanol. This corroborates the finding by Petruccelli et al. (2016). In contrast, *dumb* and *damb* males defective in dDA1 (D1) and DAMB (D5) receptors, respectively, exhibited normal sensitivity (*p* > 0.05, Figure 1B). When MSTs of *dumb*, *damb* and *der* males were examined during daily ethanol exposures, all mutants developed tolerance similar to *Canton-S* (*Canton-S*: *F*_(3,101)_ = 35.9762, *p* < 0.0001; der: *F*_(3,90)_ = 7.4871, *p* = 0.0002; *dumb*^1^, *p* < 0.001; *dumb*^2^, *p* < 0.0001; *p* < 0.0001, *damb*; Figures 1C,D). This indicates that D1 family receptors are not important for tolerance to the sedative effect of ethanol.

### Behavioral Sensitization to the Disinhibition Effect of Ethanol

**Figure 2:**
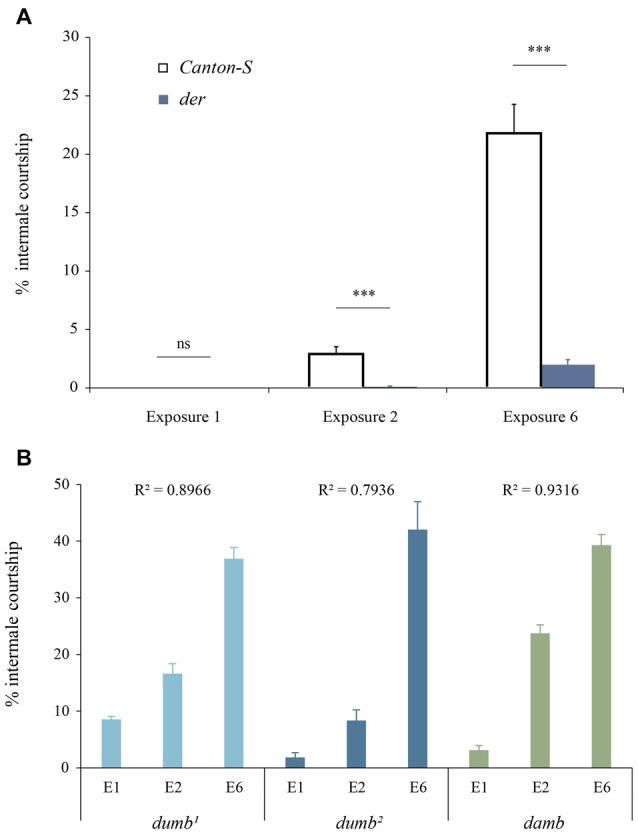
*der* mutant males exhibit impaired sensitization to the disinhibition effect of ethanol. **(A)** *der* mutant males showed significantly reduced disinhibited courtship compared to the control *Canton-S* on the 2nd (exposure 2) and 6th (exposure 6) day of daily ethanol exposure (***p < 0.0001 by ANOVA and *post hoc* Tukey-Kramer HSD tests; *Canton-S*, *n* = 17; *der*, *n* = 19). ns, not significant. **(B)** dDA1 receptor mutants *dumb*^1^ and *dumb*^2^ as well as DAMB receptor mutant *damb* developed normal behavioral sensitization to the ethanol-induced disinhibition. (*dumb*^1^, *p* < 0.0001, *n* = 7; *dumb*^2^, *p* < 0.0001, *n* = 7; *damb*, *p* < 0.0001, *n* = 6). E, exposure.

*Drosophila* males typically court females and rarely court males. Under daily ethanol exposure, however, *Canton-S* males display the escalated levels of intermale courtship (*R*^2^ = 0.7289, *F*_(2,48)_ = 64.5414, *p* < 0.0001; Figure 2A), which require dopamine neuronal activity (Lee et al., 2008). To explore the mechanism by which dopamine regulates behavioral disinhibition and sensitization, we examined the D1 family receptor mutants’ courtship behavior under the influence of ethanol. Both *dumb* and *damb* males developed behavioral sensitization to the disinhibition effect of ethanol (*dumb*^1^: *R*^2^ = 0.8966, *F*_(3,24)_ = 69.3456, *p* < 0.0001; *dumb*^2^: *R*^2^ = 0.7936, *F*_(3,24)_ = 30.7652, *p* < 0.0001; *damb*: *R*^2^ = 0.9316, *F*_(3,20)_ = 90.8291, *p* < 0.0001; Figure 2B). *der* males, on the other hand, exhibited the substantially reduced levels of intermale courtship on all exposures compared to *Canton-S* males (*p* < 0.0001; Figure 2A). This suggests that DopEcR is required for behavioral sensitization to the disinhibition effect of ethanol.

### Neural Substrate for Behavioral Sensitization

**Figure 3:**
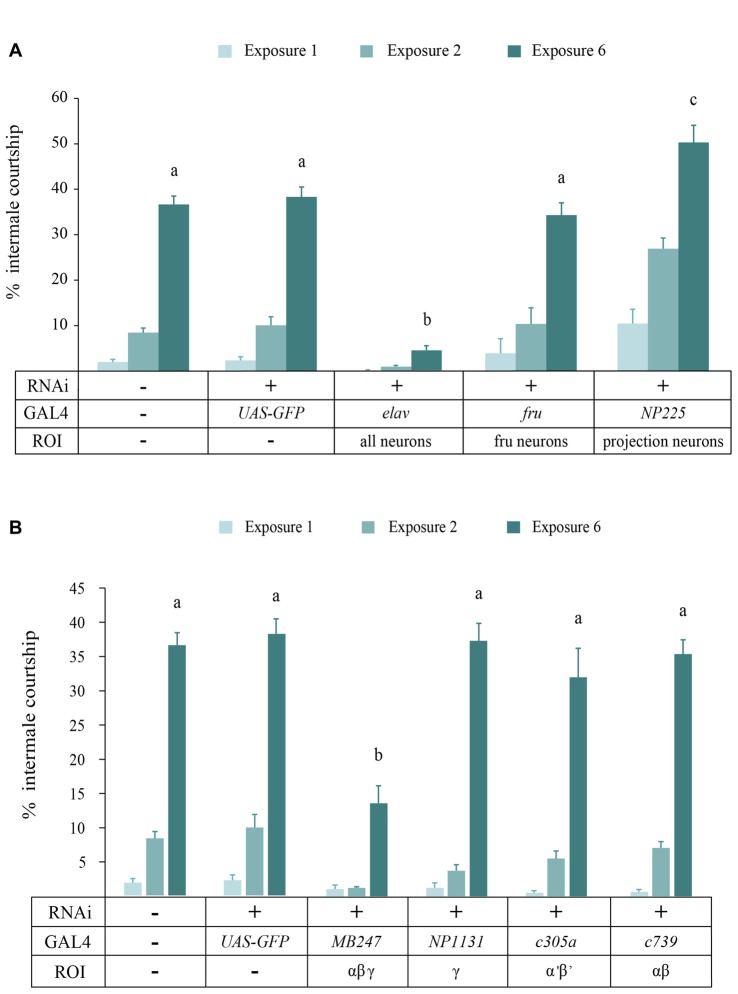
DopEcR knockdown in the mushroom body (MB) αβ and γ neurons suppresses sensitization. **(A)** Pan-neuronal DopEcR knockdown (*elav-GAL4/+;UAS-DopEcR-RNAi/+*, *R*^2^ = 0.50, *n* = 6) led to substantially reduced sensitization compared to *Canton-S* or the transgenic control (*UAS-GFP/+;UAS-DopEcR-RNAi/+*, *R*^2^ = 0.91, *n* = 6). Different letters on the bars (i.e., a, b and c) denote significant difference when all genotypes on exposure 6 were compared (ANOVA, *p* < 0.0001). Normal behavioral sensitization was observed when DopEcR was knocked down in the *fruitless* (*fru*) neurons (*fru-GAL4/+;UAS-DopEcR-RNAi/+*, *R*^2^ = 0.70, *n* = 5). DopEcR knockdown in the projection neurons resulted in slightly increased sensitization (*NP225-GAL4/+;UAS-DopEcR-RNAi/+*, *R*^2^ = 0.75, *n* = 7) (c, *p* = 0.0186 compared to the transgenic control by *post hoc* Dunnett’s test). **(B)** DopEcR knockdown in the MB α, β and γ neurons (*MB247-GAL4/+;UAS-DopEcR-RNAi/+*, *R*^2^ = 0.61, *n* = 7) led to significant reduction in behavioral sensitization (*p* < 0.0001). DopEcR knockdown in individual MB subsets (αβ, *c739/+;UAS-DopEcR-RNAi/+*, *R*^2^ = 0.95, *n* = 6; α’β’, *c305a/+;UAS-DopEcR-RNAi/+*, *R*^2^ = 0.81, *n* = 6; γ, *NP1131/+;UAS-DopEcR-RNAi/+*, *R*^2^ = 0.93, *n* = 6) resulted in normal sensitization.

To identify the neural structure where DopEcR regulates behavioral sensitization, we employed the GAL4/UAS binary system and RNA interference (RNAi) for cell type-specific knockdown of DopEcR expression. In this study we used an additional control line carrying *UAS-GFP* and *UAS-DopEcR RNAi* since courtship behavior could be sensitive to the *mw* in a transgenic construct (Lee et al., 2008). To establish effectiveness of DopEcR RNAi, we used the pan-neuronal driver *elav-GAL4* to express double-stranded DopEcR RNA for RNAi in all neurons. Like *der* mutants, the flies with pan neuronal DopEcR knockdown showed severe impairment in behavioral sensitization (*p* < 0.0001; Figure 3A). We reasoned that the neural substrate for the DopEcR’s function in behavioral sensitization could be the neurons regulating courtship behavior or high order brain structures mediating learning and memory. Fruitless-expressing neurons control male courtship behavior (Manoli et al., 2005; Stockinger et al., 2005) thus represent a potential neural site for the DopEcR’s function. The projection neurons are another candidate for the DopEcR’s function because they have dendrites in the antennal lobes and axons at the lateral horn and the MB calyx that are high order brain centers for pheromone information processing, learning and memory (Thum et al., 2007; Grosjean et al., 2011). When DopEcR was knocked down in Fruitless neurons, we did not observe a significant change in behavioral sensitization (*p* > 0.05; *fru-GAL4* in Figure 3A) while DopEcR knockdown in the projection neurons resulted in slightly increased sensitization (*p* = 0.0186; *NP225-GAL4*). Above all, we observed markedly reduced sensitization in the flies with DopEcR knockdown in the MB neurons (*p* < 0.0001; *MB247-GAL4* in Figure 3B). The MB consists of αβ, α’β’ and γ neurons where *MB247-GAL4* is expressed in αβ and γ neurons. We next asked whether DopEcR in each MB substructure is sufficient for behavioral sensitization. When DopEcR RNAi was induced only in αβ, α’β’ or γ neurons via the *c739*-, *c305a*- or *NP1131-GAL4* driver, respectively, the flies developed normal behavioral sensitization (*p* > 0.05). This suggests that DopEcR in the αβ and γ, but not αβ or γ alone, is needed for behavioral sensitization to the disinhibition effect of ethanol.

### Temporal Requirement for DopEcR Function

**Figure 4:**
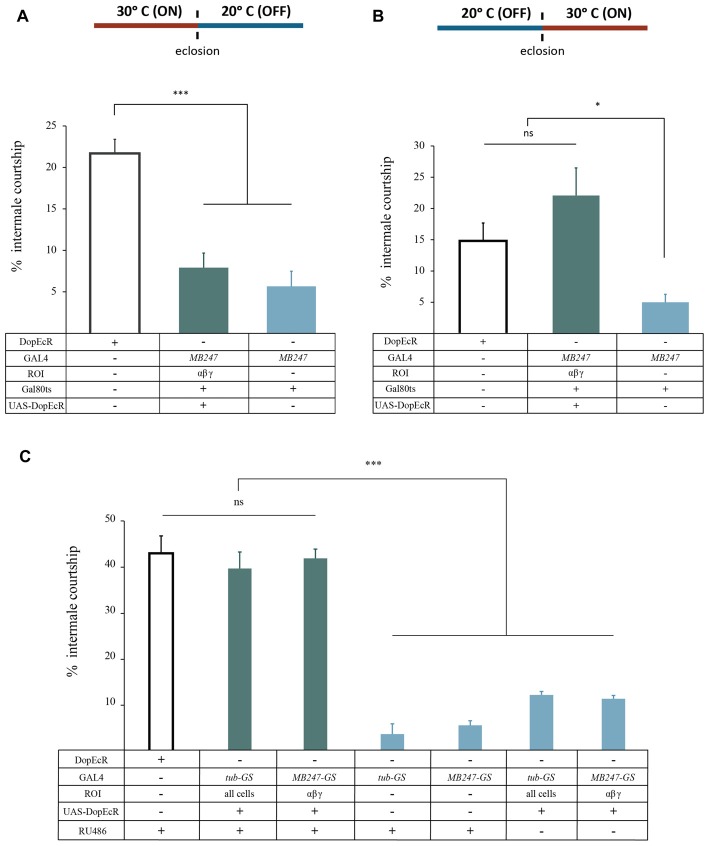
DopEcR is needed during adulthood to mediate sensitization. The *der* mutant males carrying *tub-GAL80*^ts^, *MB247-GAL4* and *UAS-DopEcR cDNA* were reared at 30°C before eclosion to induce DopEcR expression during development **(A)** or after eclosion to induce DopEcR expression during adulthood **(B)**. **(A)** The *der* males with reinstated DopEcR expression only during development (*MB247, GAL80*^ts^*/UAS-DopEcR cDNA;der*, *n* = 7) exhibited behavioral sensitization at the level comparable to that of the *der* transgenic mutant (*MB247, GAL80*^ts^/+;*der*; *p* > 0.05, *n* = 6) but lower than that of the *Canton-S* control (***p < 0.0001, *n* = 6). **(B)** The *der* males with reinstated DopEcR expression only during adulthood (*MB247, GAL80* ^ts^*/UAS-DopEcR cDNA;der*; *n* = 7) showed behavioral sensitization comparable to the control (ns, *p* > 0.05, *n* = 7) but higher than the *der* mutant (*MB247, GAL80*^ts^/+;*der*; *p < 0.05, *n* = 4). **(C)** The *der* males carrying *UAS-DopEcR-cDNA* and either *tub-GS-GAL4* (*UAS-DopEcR cDNA/+;tub-GS-GAL4, der/der*, *R*^2^ = 0.8486, *n* = 7) or *MB247-GS-GAL4* (*UAS-DopEcR cDNA /+;MB247-GS-GAL4, der/der*, *R*^2^ = 0.9834, *n* = 4) displayed sensitization similar to the control (*R*^2^ = 0.9113, *n* = 5) when treated with RU486 (ns, *p* > 0.05), but significantly higher than the *der* mutant controls (*tub-GS-GAL4, der/der*, *n* = 4; *MB247-GS-GAL4, der/der*, *n* = 4) treated with RU486 or the *der* mutants carrying the rescue transgenes without RU486 treatment (***p < 0.0001). The percent intermale courtship on the exposure 6 are shown. ns, not significant.

DopEcR is expressed throughout development and adulthood (FlyBase Consortium, 2003; Inagaki et al., 2012; Ishimoto et al., 2013; Petruccelli et al., 2016). To test whether the sensitization phenotype is caused by developmental or physiological DopEcR deficiency, we adopted two approaches, TARGET and Gene Switch (GS) for temporally restricted reinstatement of DopEcR expression in the MB neurons of *der* mutants. TARGET (McGuire et al., 2004) is the GAL4/UAS combined with GAL80^ts^ that confers temporally restricted expression of a transgene downstream of UAS, which we used successfully in the study of dDA1 in olfactory memory formation (Kim et al., 2007). Briefly, GAL80^ts^ is active as a GAL4 repressor at 20°C but inactive at 30°C, allowing GAL4 activity thereby UAS activation. The *der* mutants carrying *tub-GAL80*^ts^, *MB247-GAL4* and *UAS-DopEcR cDNA* were reared at 30°C throughout development but maintained at 20°C right after eclosion to induce DopEcR expression only during development (Figure 4A). To induce DopEcR only during adulthood, possibly at the time of ethanol exposure, the *der* mutants carrying *tub-GAL80*^ts^, *MB247-GAL4* and *UAS-DopEcR cDNA* were reared at 20°C throughout development but maintained at 30°C 2 days after eclosion. *Canton-S* and *der* mutant carrying *tub-GAL80*^ts^ and *MB247-GAL4* but not *UAS-DopEcR* *cDNA* were treated with the same temperature manipulation to serve as controls. As shown in Figure 4A, the *der* males with DopEcR expression only during development exhibited impaired behavioral sensitization thus there was no rescue (*F*_(2,16)_ = 23.2, *p* < 0.0001). In contrast, the *der* males with DopEcR expression only during adulthood fully reinstated behavioral sensitization (*p* > 0.05 compared to *Canton-S*; Figure 4B). This indicates the role of DopEcR during adulthood for disinhibition sensitization.

We observed that the flies with the temperature manipulation displayed highly variable ethanol sensitivity and sensitization. Thus as a complementary approach, we used the GS system in which GAL4 is fused to the progesterone receptor. Only in the presence of the steroid RU486, GAL4 can activate UAS for downstream gene expression (Roman et al., 2001). We tested the *der* mutants carrying *UAS-DopEcR cDNA* and *tub-GS-GAL4* or *MB-GS-GAL4* for ubiquitous or MB expression of DopEcR, respectively, at the time of ethanol exposure. When treated with RU486, the *der* males with DopEcR expression in all cells or MB neurons displayed the level of sensitization substantially higher than that of the *der* males carrying only *tub-GS-GAL4* or *MB-GS-GAL4*, but comparable to the *Canton-S* level (*F*_(6,24)_ = 43.2375, *p* < 0.0001; Figure 4C). The *der* males carrying the same transgenes (i.e., *UAS-DopEcR-cDNA* and *tub-GS-GAL4* or *MB-GS-GAL4*) that were not fed with RU486 exhibited impaired sensitization similar to the *der* mutants carrying *tub-GS-GAL4* or *MB-GS-GAL4* (*p* > 0.05 by *post hoc* Tukey-Kramer HSD test; Figure 4C). These observations together demonstrate that DopEcR expression during adulthood is sufficient for sensitization, supporting the physiological role of DopEcR at the time of ethanol exposure for this behavioral plasticity.

### Expression Patterns of DopEcR

**Figure 5:**
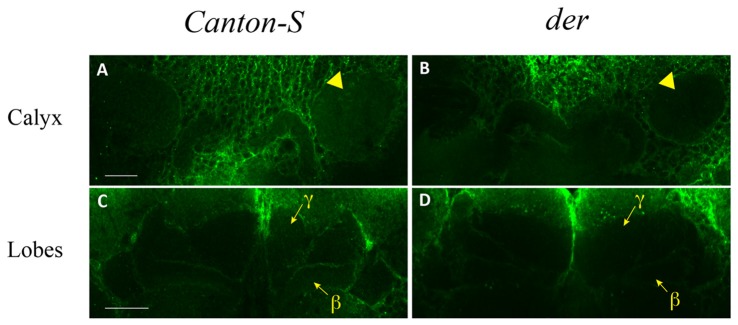
DopEcR expression in the MB neuropil. DopEcR immunoreactivity is evident in the calyx (**A**, arrowhead) and medial lobes β and γ (**C**, arrows) in the *Canton-S* brain but barely detectable in the *der* brain (**B**, arrowhead for calyx; **D**, arrows for medial lobes). One micron optical sections were made using a 40x **(A,B)** or 63x **(C,D)** objective in the confocal microscope and three sections were stacked in all images. Scale bar, 25 micron.

The study of *DopEcR enhancer-GAL4* shows that DopEcR is expressed in the MB αβ and γ neurons (Ishimoto et al., 2013). It is however unclear where DopEcR is localized in the MB. To address this, we used immunohistochemical analysis. We made the fusion construct of Glutathione S-transferase and the third cytoplasmic loop of DopEcR as we have previously characterized the dDA1 and DAMB expression patterns (Han et al., 1996, 1998). We also made the antibody against the peptide corresponding to part of the third cytoplasmic loop. The antibodies made against the fusion protein in rabbits and mice did not provide reliable staining; however, the antibody made against the peptide revealed consistent staining in the MB neuropil. It is worth mentioning that the antibody did not penetrate inside the brain under numerous conditions that we tried and also strongly stained the cell membrane of nearly all neurons and glia in both *Canton-S* and *der* (Figure 5; **Supplementary Movie Files**). Nonetheless, DopEcR immunoreactivity was clearly visible in the MB calyx (dendritic structure; Figure 5A, Supplementary Figure S1), α lobe core and β lobe (axonal structure) in the *Canton-S* brain (Figure 5C, **Supplementary Movie 1**). DopEcR immunoreactivity in the γ lobe was also detectable but at a very low level (Figure 5C and **Supplementary Movie 1**). On the contrary, DopEcR immunoreactivity in all MB neuropil was barely detectable in the *der* brain (Figures 5B,D, Supplementary Figure S1 and **Supplementary Movie 2**). These observations suggest that the site of DopEcR’s function for sensitization is the MB dendrites in the calyx or axons in the α, β or γ lobe, or both locations.

## Discussion

In this report, we show that DopEcR in the MB αβ and γ neurons mediates behavioral sensitization to the disinhibition effect of ethanol. Further, we demonstrate that the DopEcR’s function is physiological rather than developmental. As in mammals, dopamine is important for the locomotor activating and rewarding effects of ethanol in flies (Bainton et al., 2000; Kong et al., 2010; Kaun et al., 2011). The D1 receptor dDA1/DopR in the ellipsoid body is involved in the locomotor stimulant effect (Kong et al., 2010) while the dopamine receptor mediating the rewarding effect is unknown. We have noted that the flies deficient in dDA1 or DAMB display augmented disinhibition on all ethanol exposures tested, and we are currently following up on this finding. These observations together indicate that dDA1 is involved in diverse effects of ethanol possibly through distinct neural circuits.

Kaun et al. (2011) examined the rewarding property of ethanol using a conditioned preference assay. They have found that all MB subsets are important for conditioned preference to the cue associated with ethanol. It has been postulated that the dopamine signal to the MB αβ lobe is crucial for preference expression (Kaun et al., 2011). Behavioral sensitization represents a form of learning and memory (Camarini and Pautassi, 2016). The neural substrate that we identified for DopEcR’s function in sensitization is consistent with the MB’s role in learning and memory as opposed to simple sensory information processing. We have previously shown that the dDA1 receptor in the MB αβ and γ neurons mediates reward memory of sucrose (Kim et al., 2007) but it is not needed for behavioral sensitization (this study). Thus, the MB αβ and γ neurons process the reinforcing effects of the natural substance sucrose and the addictive drug ethanol via distinct dopamine receptors dDA1 and DopEcR, respectively.

DopEcR responds to dopamine as well as the steroid hormone ecdysone (Srivastava et al., 2005). For short-term memory in courtship conditioning and the sedative effect of ethanol, ecdysone is as a major ligand for DopEcR (Ishimoto et al., 2013; Petruccelli et al., 2016). Dopamine, on the other hand, activates DopEcR in the gustatory receptor neurons to enhance sensitivity to sugar in hungry flies (Inagaki et al., 2012). In male moths, DopEcR in the antennal lobe regulates behavioral responses to pheromones, which require both dopamine and ecdysone as ligands (Abrieux et al., 2013, 2014). We show that both dopamine neurotransmission blockade (Lee et al., 2008) and DopEcR deficiency (this study) cause severely impaired behavioral sensitization, implicating dopamine as a major ligand for the DopEcR function. This notion is supported by the recent study (Chen et al., 2017) demonstrating that the increased level of dopamine in the PPL2ab neurons enhances intermale courtship. The PPL2ab neurons innervate the MB calyx (Mao and Davis, 2009) where DopEcR is localized (Figure 5A). It remains to be clarified, nevertheless, whether dopamine or both dopamine and ecdysone together act on DopEcR for behavioral sensitization to the ethanol’s effect on courtship disinhibition.

Dopamine is a key neuromodulator mediating not only reward and pleasure associated with natural stimuli and addictive substances but also neuroadaptations underlying abuse and addiction (Clarke and Adermark, 2015; Volkow and Morales, 2015; Camarini and Pautassi, 2016). Behavioral sensitization is widely studied as a model for drug addiction and typically measured to the locomotor-stimulant effect of alcohol and other drugs (Berridge and Robinson, 2016). Enhanced disinhibition and impulsivity induced by ethanol contribute to risky behaviors such as sexual assaults, aggression and drug seeking or abuse (Field et al., 2010; Dalley et al., 2011; Morris et al., 2016), all of which negatively impact our society. However, the underlying mechanism still remains poorly understood. The study reported here may help narrow the knowledge gap. On this line of thought, GPR30/GPER1 represents the membrane G-protein coupled receptor that mediates non-genomic actions of the steroid hormone estrogen in mammals (Maggiolini and Picard, 2010). When tested *in vitro*, GPR30 responds to dopamine in a dose-dependent manner to increase cAMP similar to DopEcR (Evans et al., 2014, 2016). GPR30’s function in ethanol-induced behaviors is unknown but it plays a crucial role in sexual motivation of male rats (Hawley et al., 2017). It would be of interest to learn whether GPR30 mediates ethanol-induced disinhibition and sensitization similar to DopEcR.

## Author Contributions

K-AH conceived and designed the experiments. GPA, SJH, PRS and PDE performed the experiments. K-AH, GPA, SJH, and PRS analyzed the data. GPA and K-AH wrote the article.

## Conflict of Interest Statement

The authors declare that the research was conducted in the absence of any commercial or financial relationships that could be construed as a potential conflict of interest.

## Supplementary Material

**Supplementary Movie 1 (CS) and 2 (der) 1**. The *Canton-S* (CS; Movie 1) and *der* (Movie 2) brains stained with the DopEcR antibody were scanned using the confocal microscope with a 20× objective, and the optical sections made every micron were stacked to create the movie files. Available online at: http://datarepo.bioinformatics.utep.edu/getdata?acc=SHF53E1DZOVXOB1.

FIGURE S1 Shown are the posterior areas of the *Canton-S* (top) and *der* (bottom) brains immunostained with the anti-DopEcR antibody. The optical sections were made every micron with a 20X objective and two sections were stacked. The calyx area on the right hemisphere in each brain is marked by arrowheads. Scale bar, 25 micron.
