# Supplementary figures and images for: Behavioral Sensitization to the Disinhibition Effect of Ethanol Requires the Dopamine/Ecdysone Receptor in Drosophila
Source: Front Syst Neurosci. 2017 Aug 2;11:56. doi: 10.3389/fnsys.2017.00056 (PMC5539124; doi:10.3389/fnsys.2017.00056)

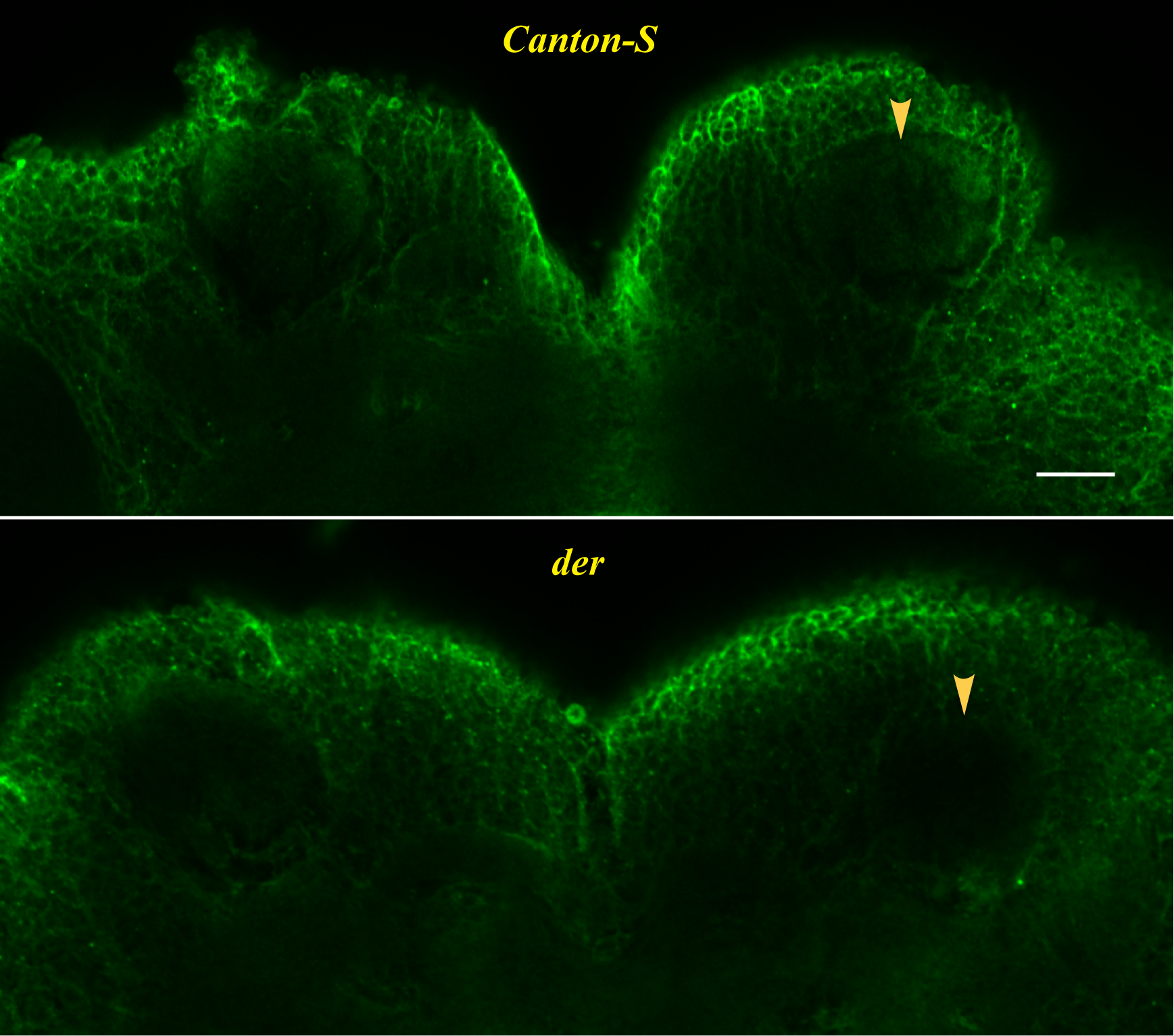

Supplement: FIGURE S1 — Shown are the posterior areas of the Canton-S (top) and der (bottom) brains immunostained with the anti-DopEcR antibody. The optical sections were made every micron with a 20X objective and two sections were stacked. The calyx area on the right hemisphere in each brain is marked by arrowheads. Scale bar, 25 micron. [file Image_1.tif]
